# Observing single nanoparticle events at the orifice of a nanopipet†
†Electronic supplementary information (ESI) available: Experimental details, zeta potential distribution, finite-element simulations, CLSM, and equation derivation. See DOI: 10.1039/c6sc02241c



**DOI:** 10.1039/c6sc02241c

**Published:** 2016-07-04

**Authors:** Ting Li, Xiulan He, Kailin Zhang, Kai Wang, Ping Yu, Lanqun Mao

**Affiliations:** a Beijing National Laboratory for Molecular Sciences , Key Laboratory of Analytical Chemistry for Living Biosystems , Institute of Chemistry , The Chinese Academy of Sciences , Beijing 100190 , China; b University of Chinese Academy of Sciences , Beijing 100049 , P. R. China . Email: lqmao@iccas.ac.cn ; Email: yuping@iccas.ac.cn ; Fax: +86-10-62559373

## Abstract

Single nanoparticle (NP) events are successfully observed at the orifice of a nanopipet by blocking the ionic current with a single NP. In addition to the traditional translocation events, we observe both staircase and blip current transients by controlling the radius ratio of NPs to nanopipet or bias potential. Confocal fluorescence microscopy and finite element simulation are used to simultaneously monitor and quantitatively understand these events, respectively. The frequency of the staircase and blip events is proportional to the NP concentration, and could be used for the quantification of NPs. This study offers a new method for NP determination and single NP behavior study.

## Abstract 2

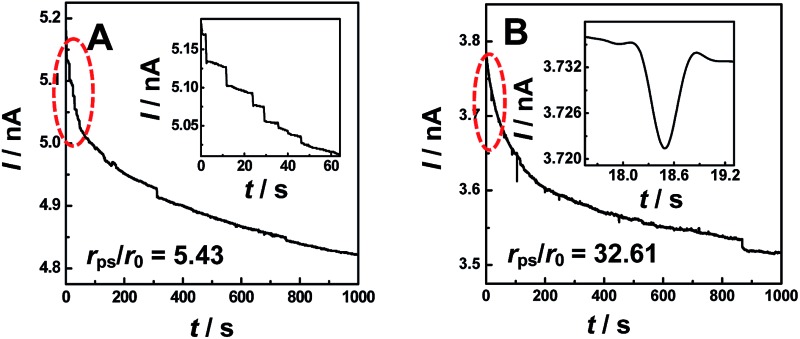
Single nanoparticle (NP) events are successfully observed at the orifice of a nanopipet by blocking the ionic current with a single NP.

Nanoparticles (NPs) including artificially synthetic (*e.g.*, metal, metal oxide and polymers) and naturally synthetic ones (*e.g.*, proteins, viruses, and vesicles) have attracted increasing attention because of their enormous applications both in fundamental studies and practical applications.^1^ As is well documented, the intrinsic features of single NPs stemming from their size, charge, shape and surface chemistry normally determine their unique functions.^2^ The classic techniques for NP characterization, such as transmission electron microscopy (TEM) and dynamic light scattering (DLS), are difficult to provide enough information closely related to functions. For instance, although TEM has been widely used for characterization of particles in a solid and dried state, it is difficult for particles in a solution phase. DLS provides information for the entire samples, but remains difficult for individual particles.^3^ Therefore, it is imperative to develop new methods for detecting and sizing individual NPs in bulk solution.

Among all techniques employed for individual particle analysis in solution, electrochemical methods have attracted much attention due to their high time resolution and good sensitivity. So far, two main electrochemical principles have been developed to analyze the behavior of individual NPs in a colloidal dispersion. One is based on the resistive-pulse technique for counting and sizing the NPs using a small aperture.^3b,4^ The other technique is based on the collision between NPs and microelectrodes,^5^ which has been coupled with different signal output modes^6^ to detect single NPs ranging from hard metals to soft particles.^7^ Very recently, this technique has also been successfully used for intracellular vesicle electrochemical cytometry.^8^ However, it is still highly desirable to develop new electrochemical principles for single NP analysis since the former principle is susceptible to pore blockage while the later one is limited to the redox properties of NPs or the additional use of electrochemical probes.

Herein, we report a new electrochemical approach utilizing a nanopipet as a reliable and robust platform for studying the individual NP behavior and detecting NPs in solution by using polystyrene (PS) particles as a model. The rationale for our technique is essentially based on blocking the ionic current with NPs when NPs are approaching at the proximity of the orifice of a nanopipet under the driving force of the electric field. When the radius of the PS particles (*r*_ps_) is larger than that of the nanopipet (*r*_0_), we interestingly observed two different current transients, as shown in Scheme 1. When *r*_ps_ is a little larger than *r*_0_ (defined as *r*_ps_ > *r*_0_), a staircase current decrease for *capture and hold* is observed (Scheme 1A). When *r*_ps_ is much larger than *r*_0_ (*i.e.*, *r*_ps_ ≫ *r*_0_), a symmetric current blip for *collision and departure* is observed (Scheme 1B). Furthermore, we find that the frequency of the staircase and blip is directly proportional to the particle concentration, and thus our findings demonstrated here could be developed into a new technique for NP analysis and single particle behavior study.

**Scheme 1:**
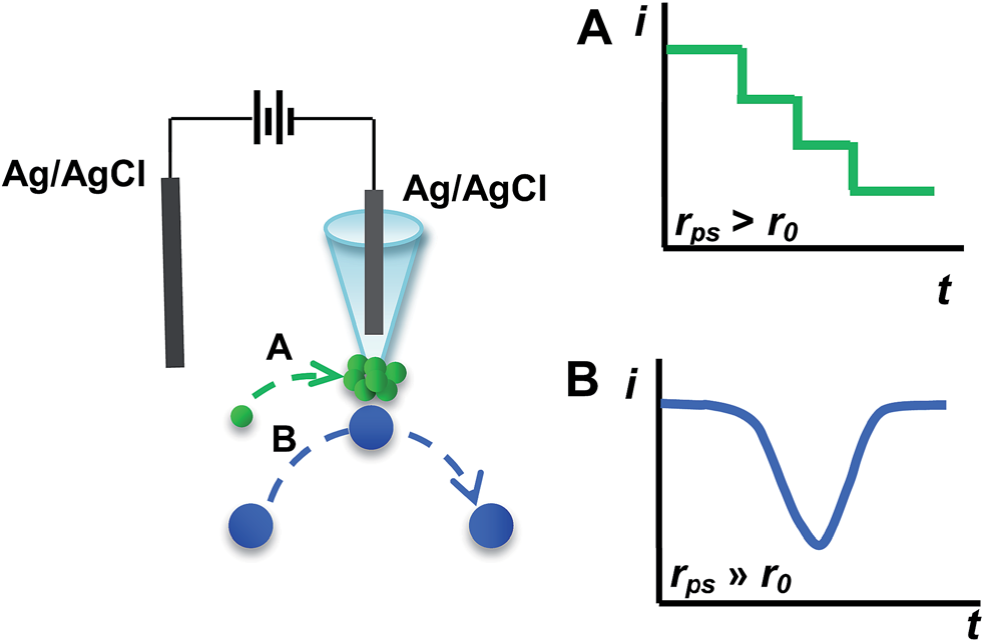
Schematic illustration of two typical models for single particle events with different radius ratios of PS (*r*_ps_) to nanopipet (*r*_0_): (A) a staircase current decrease for *capture and hold* (*r*_ps_ > *r*_0_), and (B) a symmetric current blip (or spike) for *collision and departure* (*r*_ps_ ≫ *r*_0_).

Fig. 1 shows typical *i*–*t* traces obtained at nanopipets with an average radius of 69 nm in 0.1 M KCl solution containing PS particles of different sizes. For the 375 nm-radius particles (*i.e.*, *r*_ps_/*r*_0_ = 5.43), the current exhibits a staircase-shaped decrease (Fig. 1A), which was attributed to the PS particles arriving at the orifice one-by-one. When *r*_ps_ was further increased to 2.25 μm (*i.e.*, *r*_ps_/*r*_0_ = 32.61), the symmetric blip current transient was observed (Fig. 1B). Moreover, we also observed these two different current transients for the same sized PS particles by changing the radius of the nanopipet (Fig. S2†). These results essentially suggest that the current transient behavior is strongly dependent on the *r*_ps_/*r*_0_ value, as schematically illustrated in Scheme 1.

**Fig. 1:**
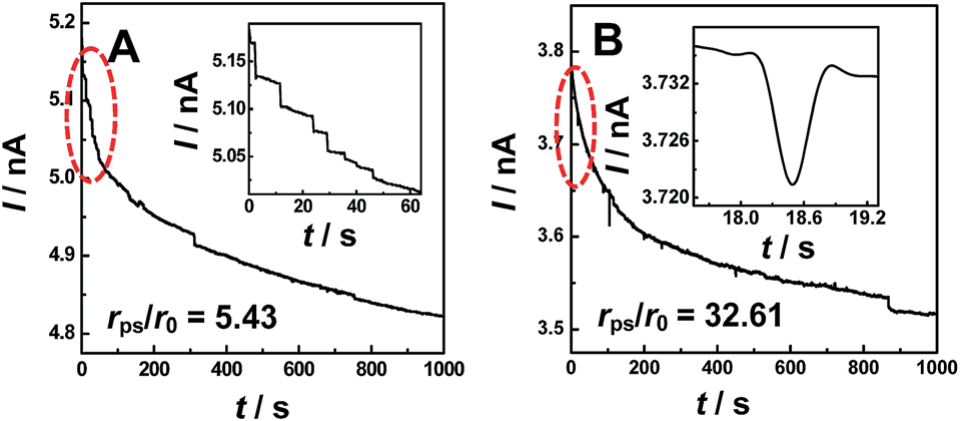
Typical *i*–*t* traces obtained at nanopipets with a radius (*r*_0_) of 69 nm in 0.1 M KCl solution containing PS particles with different radii (*r*_ps_). (A) *r*_ps_ = 375 nm, *C*_ps_ = 0.59 pM; and (B) *r*_ps_ = 2.25 μm, *C*_ps_ = 16.9 fM. Insets: amplified current transients indicated with red circles in the figures. The applied potential was 200 mV. The data acquisition time was 10 ms.

For the electrochemical systems based on nanochannels such as nanopipet and nanopore, the potential drop occurs near the orifice, where the resistance is the largest.^9^ In this case, the ionic current would rapidly decrease when the NPs are positioned in the channel under the driving force of the electric field.^10^ However, when the radius of the NPs is larger than that of the nanopipet and consequently cannot enter into the nanopipet, the current transients had never been reported and such current transients were observed in this study for the first time, as typically shown in Fig. 1A and B. To understand such current transients, we performed electric-field distribution simulation around the orifice (for more details, ESI S4†), and the results show that the strongest electric field was distributed near the orifice of the nanopipet, both inside and outside of the orifice, demonstrating that the electric field could be extended to the outer side of the nanopipet (Fig. S3†). This phenomenon was also previously observed by White *et al.* ^11^ The external electric field essentially leads to the current transients when the size of the particles was larger than that of the nanopipet (Fig. 1A and B), and these current transients actually originated from the blocked ionic current when the NPs arrive near the orifice of the nanopipet. Moreover, the results of finite element simulation suggest that positioning the particles at the opening of the nanopipet would cause a decrease of the current (Fig. S4†).

To further confirm the correlation between these two current transients (Fig. 1A and B) and the positions of particles experimentally, we simultaneously conducted confocal laser scanning microscopy and electrochemical measurements (ESI S5†). The synchronous electrochemical and optical experiments revealed that the staircase current actually resulted from the capture of the PS particle at the orifice of the nanopipet (Fig. S5†), and the multiple staircases were consequently observed due to the continuous capture of the particles at the orifice of the nanopipet (Fig. S6†). Meanwhile, the symmetric blip transient current was resulted from the *collision and departure* of the NPs (Fig. S7†). These results directly confirmed the models that we proposed in Scheme 1.

To investigate the origin of the current transients, the driving force was first estimated. Under the present conditions, convection could be ignorable since no stirring was applied during the experiments. Therefore, the PS particles could approach the orifice of the nanopipet *via* diffusion and migration. The migration rate (*f*_mig_) for the NPs to arrive at the orifice is a consequence of the electric field in bulk solution and could be given in a good approximation in eqn (1):1
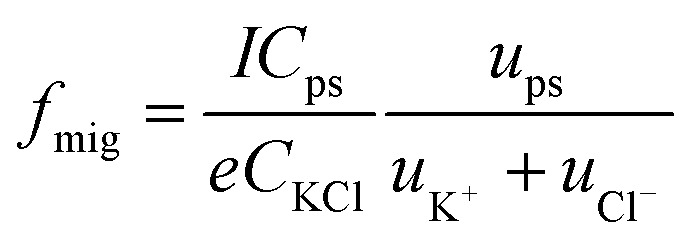
where *I* is the ionic current across the nanopipet, and *e* is the unit of charge. *C* and *u* refer to the concentration and electrophoretic mobility, respectively. Details for the derivation of eqn (1) are provided in ESI (S6†).

The diffusion rate (*f*_dif_) for pipet was estimated using eqn (2):^12^

2 *f*_dif_ = 3.35π*D*_ps_*C*_ps_*r*_0_*N*_A_

where, *D*_ps_, *C*_ps_, and *r*_0_ are the diffusion coefficient of the particles, the concentration of the particles, and the radius of the nanopipet, respectively.

Under the conditions in Fig. 1A and B, *f*_mig_ was estimated as 0.048 s^–1^ for 375 nm-radius PS, and 0.0008 s^–1^ for 2.25 μm-radius PS particles. The diffusion rates were estimated to be 1.68 × 10^–4^ s^–1^ and 8.16 × 10^–7^ s^–1^, respectively, which were insignificant as compared to those of migration.^13^ In addition, the event frequency counted experimentally from Fig. 1A and B are 0.023 s^–1^ and 0.006 s^–1^, respectively, which were consistent with those estimated using eqn (1). These results substantially suggest the dominant role of migration in the models (Scheme 1). This conclusion was further confirmed by the absence of the current transients when the sign of the applied potential was reversed (Fig. S8†).

Since migration controls the arrival rate of the particles, the effect of the applied potential on current transients was further investigated. Fig. 2 shows partially enlarged *i*–*t* traces obtained at a 69 nm-radius nanopipet in 0.1 M KCl solution containing 375 nm-radius (A) and 2.25 μm-radius (B) PS particles at various applied potentials (original data as shown in Fig. S9†). For both 375 nm-radius (Fig. 2A) and 2.25 μm-radius (Fig. 2B) particles, the current transient was interestingly changed from blip (0.05 V, red curves) to staircase (0.5 V, blue curves) with increasing applied potential. Differently, for 375 nm-radius PS particles (Fig. 2A), the staircase current transients were observed at the potential of 0.2 V (Fig. 2A, black curve), but the blip current transients were observed at the same potential for the particles with larger radius (*e.g.*, 2.25 μm, Fig. 2B, black curve). These results demonstrate that the shape (*i.e.*, blip and staircase) of current transients was dependent both on the radius ratio (*i.e.*, *r*_ps_/*r*_0_) and the applied potential.

**Fig. 2:**
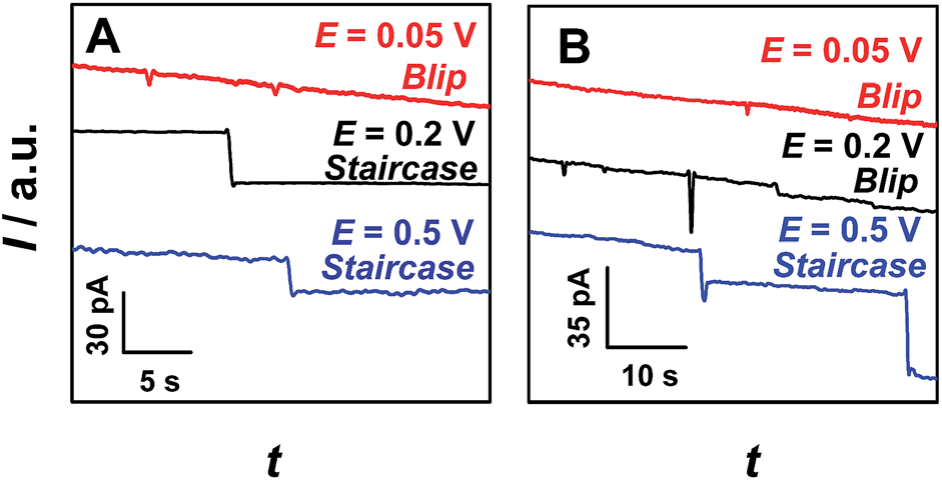
Partially enlarged *i*–*t* traces obtained at the nanopipets with a radius of 69 nm in 0.1 M KCl solution containing 0.59 pM 375 nm-radius PS particles (A) and 16.9 fM 2.25 μm-radius PS particles (B) at different applied potentials, as indicated in the figures. The data acquisition time was 10 ms.

The observation of blip or staircase current transient described above was considered to be a consequence of the balance of electric-field force controlled by the applied potential, elastic force originated from the collision, and electroosmotic force related to the charge of the nanopipet. The electric-field force draws the particles into the nanopipet, while the electroosmotic force and elastic force drive the particles out of the nanopipet. When *r*_ps_/*r*_0_ was constant, the electric-field force would increase with increasing applied potential. At low applied potentials (*e.g.*, 0.05 V, red curves in Fig. 2), the electric-field force may not be strong enough to hold the particles on the orifice and the particles would leave the orifice under the drag of elastic force and/or electroosmotic force. As a result, the blip current transients were observed. In contrast, when the applied potentials were high enough (*e.g.*, 0.5 V, blue curves in Fig. 2), the particle could be stably captured at the orifice of the nanopipet under the electric field, and thus the staircase current transients were observed. On the other hand, when the applied potential was constant, the capture part would decrease with increasing radius ratio (*i.e.*, *r*_ps_/*r*_0_), and thus the particle would easily depart from the nanopipet at large *r*_ps_/*r*_0_ values. As a consequence, the blip current was observed for a large radius ratio (*i.e.*, *r*_ps_/*r*_0_ = 32.61, Fig. 2B, black curve), but the staircase current occurs for a relatively small radius ratio (*i.e.*, *r*_ps_/*r*_0_ = 5.43, Fig. 2A, black curve). Moreover, we also think that the electroosmotic force at the opening of the nanopipet may also play an important role, the quantitative understanding of this phenomenon is currently being investigated in our group.

To explore the applicability of our observations for NP analysis, we investigated the relationship between current transient frequency and the particle concentration. We found the number of current transients (both for blip and staircase) increased with increasing PS particle concentration (Fig. S10†). Fig. 3 shows the relationship between the event frequency and the particle concentration at a constant applied potential (*i.e.*, 0.2 V). The event frequency was calculated as the counts of current transient features observed during a thousand-second period. A linear dependence was observed between the event frequency and particle concentrations both for the staircase (Fig. 3A) and the blip (Fig. 3B) current transients, demonstrating that our observation may be potentially useful in further quantitative analysis of particle concentration. Moreover, the different current transients (*i.e.*, blip and staircase) were observed with the same radius nanopipet at the same applied potential for particles with different radii (Fig. 3C and D), demonstrating that the observation in this study might be developed into a new technique for simultaneously determining two kinds of particles with different sizes.

**Fig. 3:**
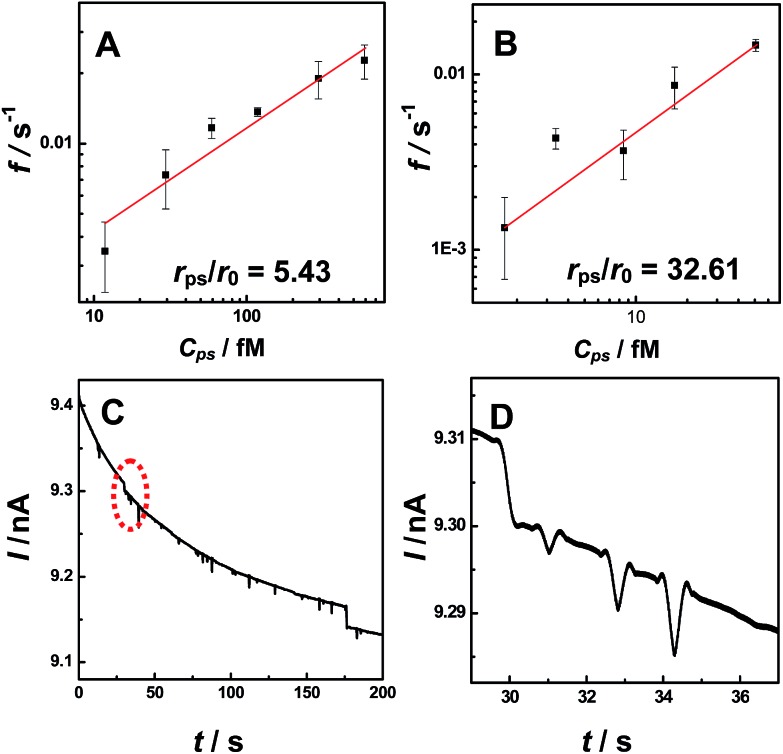
Plots of event frequency *versus* particle concentrations for staircase ((A), 375 nm-radius PS particles) and blip ((B), 2.25 μm-radius PS particles) current transient. (C) Typical *i*–*t* trace for 69 nm-radius nanopipet in 0.1 M KCl solution containing both 2.25 μm-radius (0.0357 wt%) and 375 nm-radius (0.0357 wt%) PS NPs. (D) Zoom in of the red circle in (C). The applied potential was 0.2 V. The data acquisition time was 10 ms.

## Conclusions

In conclusion, we have successfully observed single particle current transients at the orifice of a nanopipet by blocking ionic current. Both staircase and blip current responses have been observed by controlling the radius ratio of NPs to nanopipet (*r*_ps_/*r*_0_) and the applied potential. The frequency of the staircase and blip was directly proportional to the particle concentration, essentially laying the basis for developing a new technique for NP analysis and individual particle behavior study. Compared with the traditional resistive-pulse technique based on translocation, the method based on the observations in this study could provide more informative results for screening the NPs according to the current transients by using one nanopipet. More importantly, the present observations, especially for blip current transient, were much more potentially for *in situ* intracellular application. Compared with the collision method based on microelectrodes, the method demonstrated here bears the following advantages: (1) the rationale through blocking ionic current not only avoids the addition of the redox molecules but also applies to the measurements for both conductive and insulating NPs; (2) no electrochemical reaction occurs in our method with less risk in NP decomposition; and (3) the nanopipet could be easily prepared using a commercially available laser-puller machine and the contamination towards the microelectrode could easily be avoided. We believe that all of these prospects make our nanopipet-based technique particularly powerful for individual particle study.
