# Supplementary material for: Observing single nanoparticle events at the orifice of a nanopipet
Source: Chem Sci. 2016 Jul 4;7(10):6365–8. doi: 10.1039/c6sc02241c (PMC5450441; doi:10.1039/c6sc02241c)
Supplement: Supplementary file 1 [file SC-007-C6SC02241C-s001.pdf]

Electronic Supplementary Material (ESI) for Chemical Science.
This journal is © The Royal Society of Chemistry 2016

Supporting Information

Observing Single Nanoparticle Events at the Orifice of a Nanopipet

Ting Li, Xiulan He, Kailin Zhang, Kai Wang, Ping Yu*, Lanqun Mao*

Beijing National Laboratory for Molecular Sciences, Key Laboratory of Analytical Chemistry for Living Biosystems, Institute of Chemistry, the Chinese Academy of Sciences, Beijing 100190, China.

Contents

Experimental details--S1

Geometry characterization of glass nanopipet---S2

Current transients with different radius nanopipettes--
S3

Finite element simulations--S4

Synchronous electrochemical and optical experiments--S5

Derivation of equation 1--S6

I-t trace at a negative potential---S7

I-t traces at different applied potentials---S8

Current transients at different particle concentrations---S9

S1 Experimental Details

1. Chemicals and Materials

Potassium Chloride (KCl, ≥ 99.5%) was bought from Sinopharm Chemical Reagent Beijing Co., Ltd.) Different radius polystyrene (PS) particles with surface carboxylic acid (-COOH) functional group were purchased from Alfa Aesar. All the chemicals were used as received without further purification. All aqueous solutions were prepared using Milli-Q water (>18 MΩ • cm) from a Milli-pore purification system.

2. Fabrication of Glass Nanopipet

Different size pipettes were fabricated by a CO_2-laser-based pipet puller (P-2000, Sutter Instrument Co.) with borosilicate glass capillaries (1.50 mm O.D. and 1.10 mm I.D., with filament (type A); 1.50 mm O.D. and 0.86 mm I.D., without filament (type B).) from the same company. The program used for type A is shown as follows. :

(Cycle 1) heat = 325, filament = 5, velocity = 20, delay = 128, pull = 50;

(Cycle 2) heat = 350, filament = 4, velocity = 15, delay = 130, pull= 175.

The nanopipettes prepared by type A were used as pulled, with average radius 69 nm.

The program for type B:

(Cycle 1) heat = 320, filament = 4, velocity = 50, delay = 210, pull = 200.

The nanopipettes pulled through type B were polished by MF-900 microgrinder (Narishige International USA, Inc.) to achieve 3-μm-radius and 1-μm-radius pipettes.

3. Nanoparticle Characterization

ζ-potential and size measurements were performed using a Malvern Zetasizer Software v7.02 (Malvern Instruments Ltd.).

Table S1. Zeta potential and electrophoretic mobility measured by DLS in 0.1 M KCl solution.

	Zeta potential (mV)	Mobility (×10^{-8} $m^2V^{-1}s^{-1}$)
375-nm-radius PS	-49.8±1.4	3.90±0.11
2.25-μm-radius-PS	-42.0±0.4	3.20±0.16

4. Cell Configuration and Data Acquisition

Current-time responses were obtained with an electrochemical workstation (CHI-660e,

Shanghai, China). An Ag/AgCl electrode was inserted inside of the pipette, and the other Ag/AgCl electrode was placed in the external electrolyte bath. All the potentials were given in this study as internal *vs*. external. Electrochemical experiments were conducted in an aqueous solution of 0.1 M KCl as the electrolyte. Stock suspension of polystyrene particles was added to this electrolyte to yield different concentrations of particles.

S2 Geometry Characterization of Glass Nanopipet

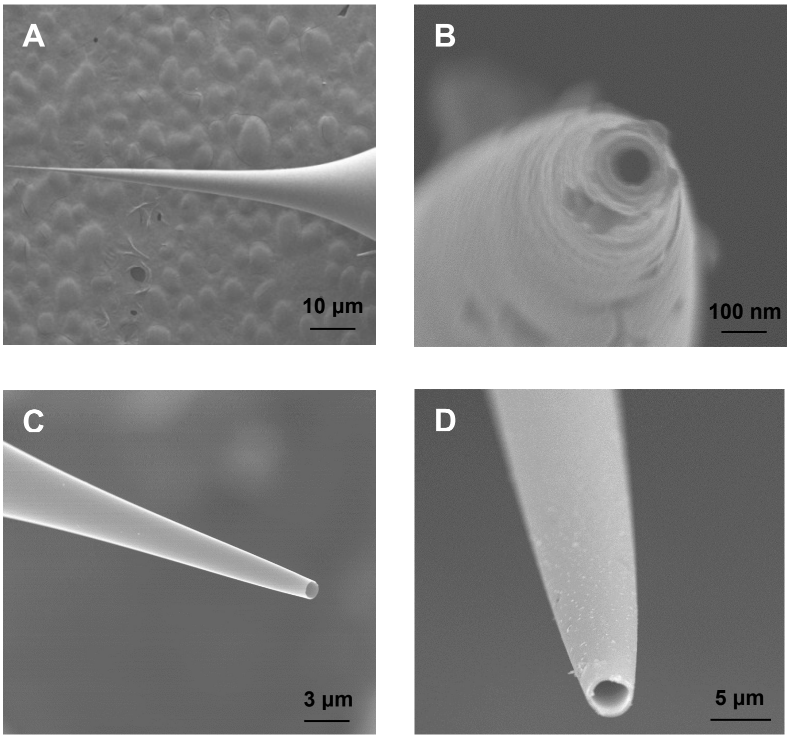

Fig. S1. SEM images of typical glass nanopipets. (A) For type A nanopipet; (B) Enlarged tip of (A); (C) and (D) For type B nanopipets of different polishing extents.

S3 Current Transients with Different Radius Nanopipets

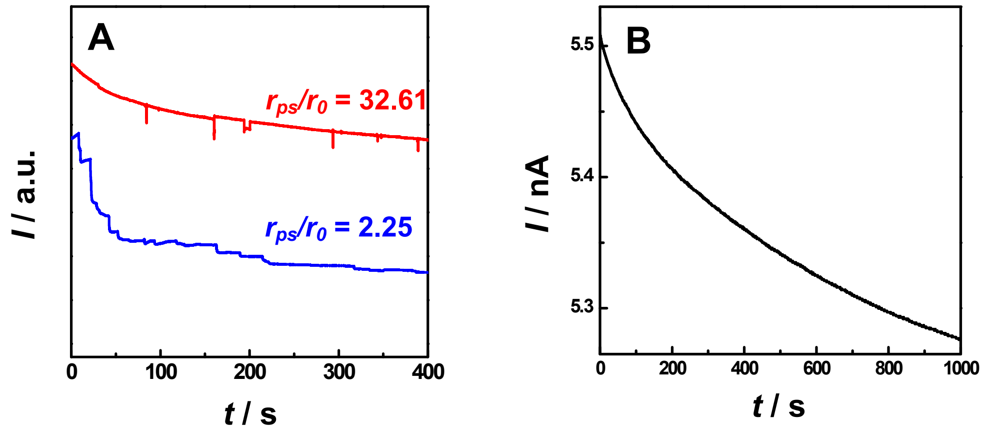

Fig. S2. (A) Typical *i-t* traces for 16.9 fM 2.25-μm-radius PS particles in 0.1 M KCl solution with different radius nanopipets: r_0 = 69 nm, r_{ps}/r_0 = 32.61(red curve); r_0 = 1 μm, r_{ps}/r_0 = 2.25 (blue curve). (B) *i-t* response in 0.1 M KCl solution in the absence of PS particles. The applied potential was 0.2 V. The data acquisition time was 10 ms.

S4 Finite-Element Simulations

The finite-element simulations were carried out with COMSOL Multiphysics 4.2a (Comsol, Inc.) using a high-performance desktop computer. Herein, the finite-element method was used to figure out the Poisson−Nernst−Planck (PNP) partial differential equations and compute the electric field distribution and current transients of particle events. The Nernst−Planck equation, equation S1, describes the fluxes of the ionic species,

$$\mathbf{J}_\mathbf{i} = -D_i \nabla c_i - \frac{z_i F}{RT} D_i c_i \nabla \mathbf{\Phi} + \mathbf{u} c_i \qquad \text{(S1)}$$

where, $\mathbf{J}_\mathrm{i}$, D_i, c_i and z_i are, respectively, the flux, diffusion coefficient, concentration and charge of species i. Φ and **u** are the local electric potential and fluid velocity, and F, R and T are the Faraday constant, the gas constant and the absolute temperature, respectively.

The relationship between the electric potential and ion concentration is described by the Poisson's equation, equation S2,

$$\nabla^2 \mathbf{\Phi} = -\frac{F}{\varepsilon} \sum_i z_i c_i \qquad \text{(S2)}$$

Here, ε is the dielectric constant of the medium.

Considering the symmetry along the centerline Z direction, half of the cross-section was used in the simulation. The number of degree of freedom was 2,521,497 for a simulation of the Poisson and Nernst-Planck without electroneutrality equations. The computation domain was assumed as potassium chloride solutions with different concentrations. The following parameters were used: T = 298 K, $D(K^+)$ = 1.957 × 10^{-9} m^2/s, $D(Cl^-)$ = 2.032 × 10^{-9} m^2/s, relative dielectric constant ε = 78, η = 1 × 10^{-3} Pa•s, and ρ = 1000 Kg/m^3. The surface charge density was defined at –1 mC/m^2. C_0 = 0.1 M. Rigorous mesh-refinement tests were performed to make sure that the solutions obtained during simulation were convergent and grid independent.

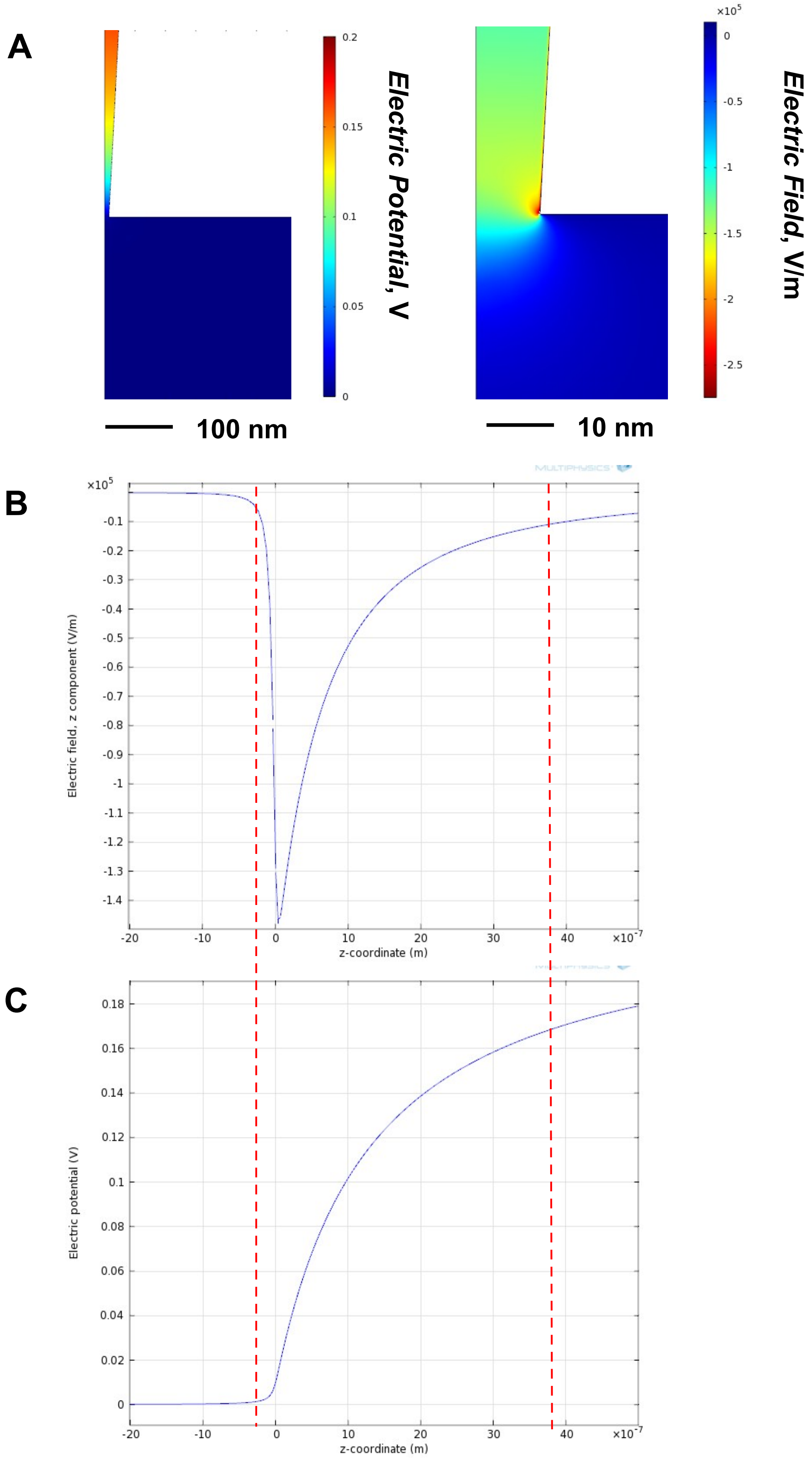
A
Electric Potential, V
100 nm
Electric Field, V/m
10 nm
B
Electric field, z component (V/m)
z-coordinate (m)
C
Electric potential (V)
z-coordinate (m)

Fig. S3. (A) Distribution of the electric potential and electric field. (B) Line profiles of the electric field and potential (C) across the sensing zone corresponding to the centerline axis of the nanopipet.

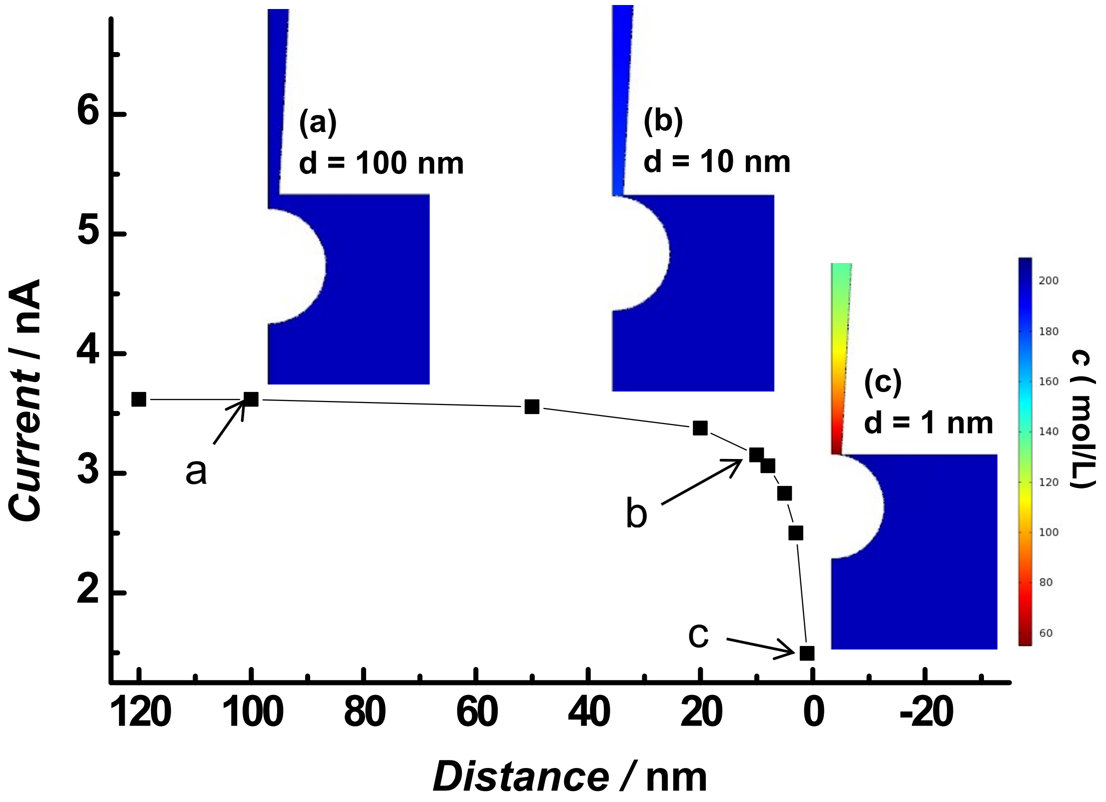

Fig. S4. Simulated current in 0.1 M KCl solution by placing a 375-nm-radius PS particle at different distance from the orifice of a 69-nm-radius nanopipet. Inset profiles represent concentration distribution of electrolyte solution at different distance from the orifice to the particle.

S5 Synchronous Electrochemical and Optical Experiments

To further confirm the correlation between two current transients (Scheme 1B and C) and the positions of particles, confocal laser scanning microscope was conducted simultaneously when the bias potential was applied across the nanopipet. Confocal laser scanning microscopy (CLSM) images were performed on an Olympus FV1000-IX81 CLSM and a Leica TCS SP confocal system (Leica, Germany). The *i-t* traces were recorded by electrochemical workstation (CHI 660e). Fluorescent dye encapsulated polystyrene NPs purchased from Alfa Aesar was used as the model.

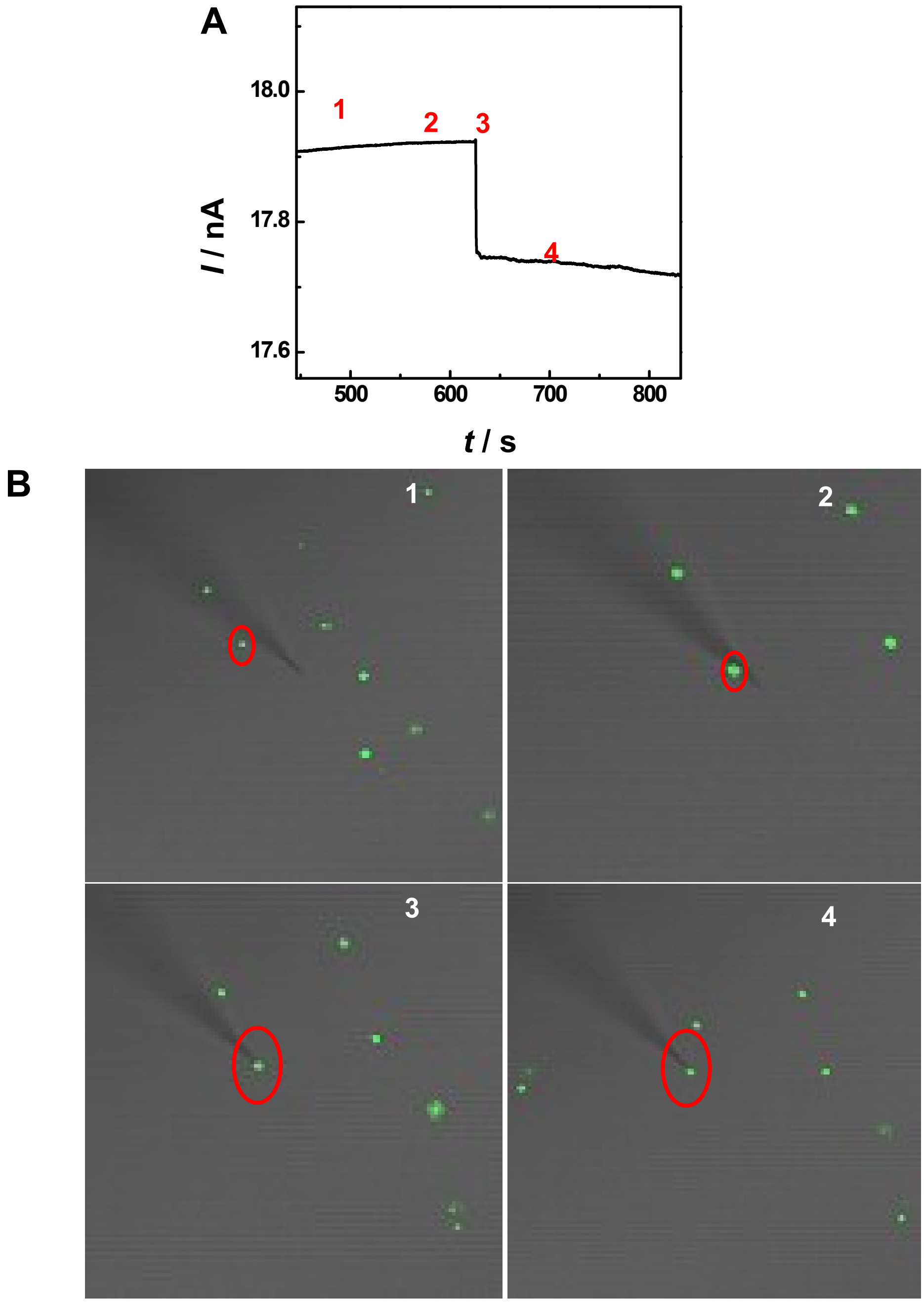

Fig. S5. (A) A typical current staircase recorded with a 69-nm-radius nanopipet in 0.1 M KCl solution containing 1-μm-radius polystyrene NPs. (B) Synchronous confocal laser

scanning micrographs (CLSM) taking at the time indicated in the (A) for one NP. The concentration of PS particles was 0.67 fM. The applied potential was 0.5 V. The data acquisition time was 10 ms.

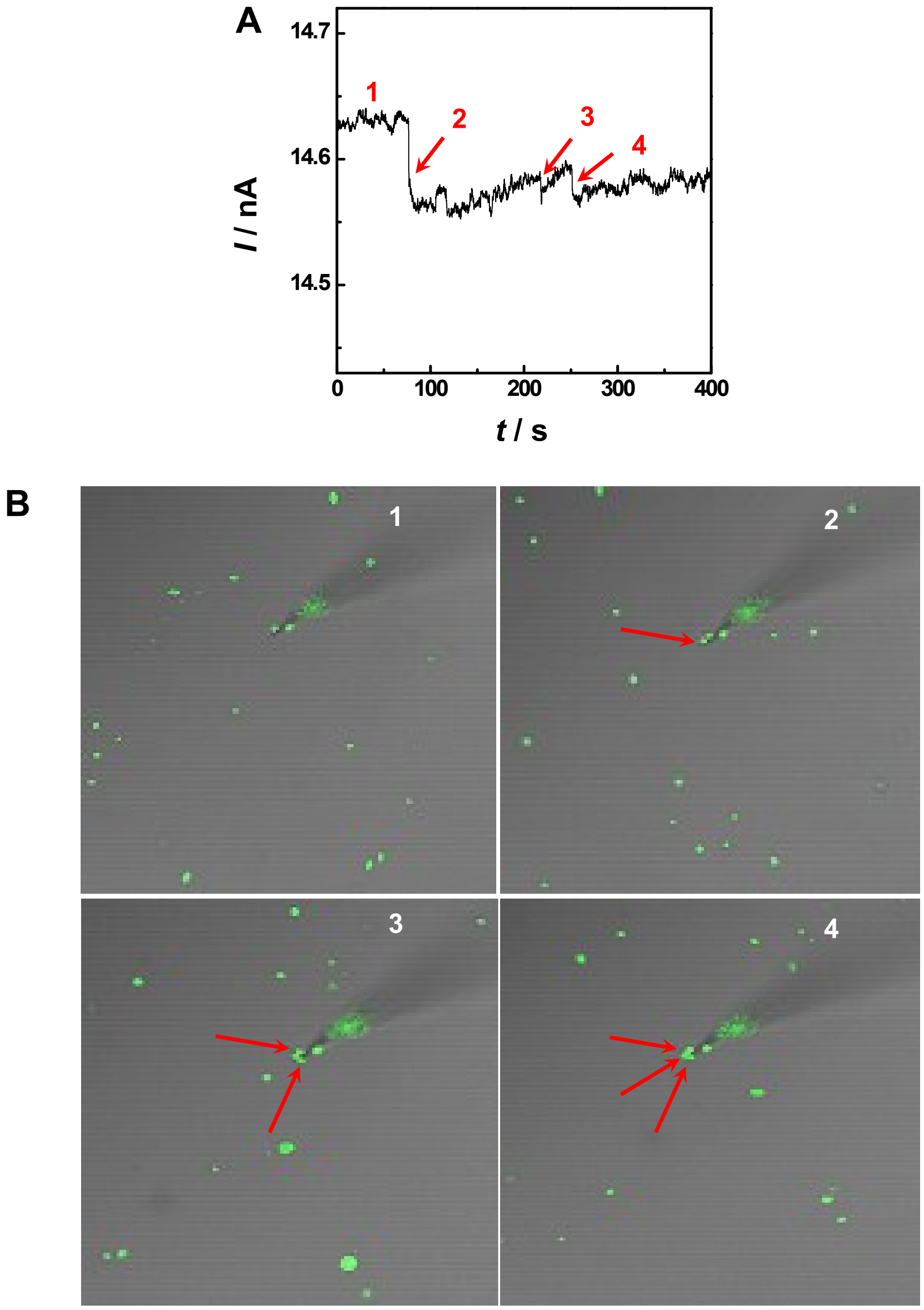

Fig. S6. (A) Representative current transient recorded with a 69-nm-radius nanopipet in 0.1 M KCl solution containing 1-μm-radius PS nanoparticles. (B) Synchronous confocal laser

scanning micrographs (CLSM) taking at the time indicated in the (A) for three different NPs. The polystyrene NPs concentration was 3.37 fM. The applied potential was 0.5 V. The data acquisition time was 10 ms.

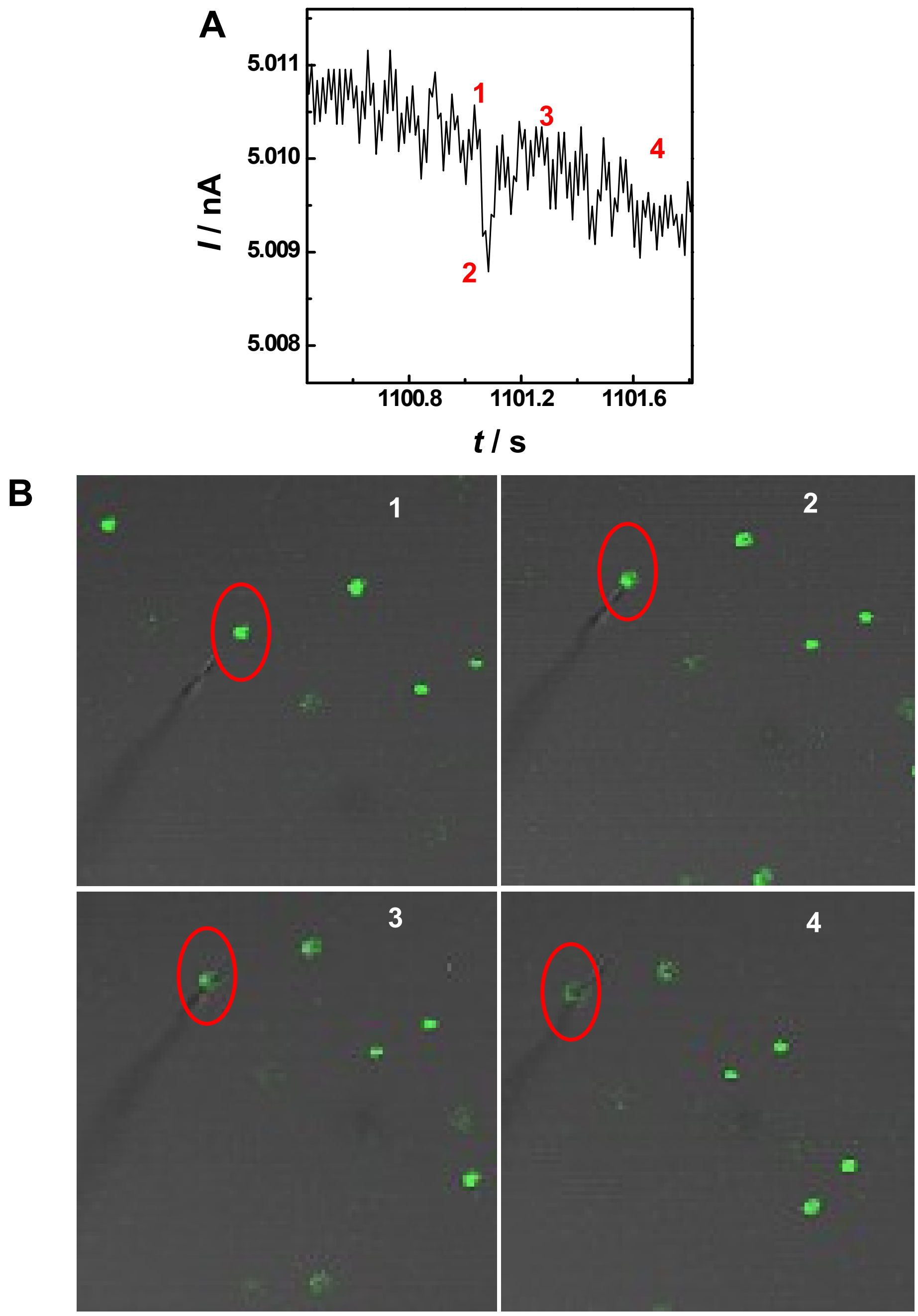

Fig. S7. (A) Typical current transient recorded with a 69-nm-radius nanopipet in 0.1 M KCl solution containing 2-μm-radius PS nanoparticles. (B) Synchronous confocal laser scanning

micrographs (CLSM) taking at the time indicated in the (A) for one NP. The polystyrene NPs concentration was 0.6 fM. Green spheres stand for polystyrene NPs encapsulating fluorescent dye. The applied potential was 0.2 V. The data acquisition time was 10 ms.

S6 Derivation of Equation 1

Equation 1 in the main text was derived as follows.

The transference number of a NP (t_{ps}) represents the relative flux of charged NPs in the electrolyte solution.

$$t_{PS} = \frac{|z_{PS}| D_{PS} C_{PS}}{\sum_i |z_i| D_i C_i} \qquad \text{(S3)}$$

The sum runs over all charged species in solution. In the present condition, this sum is completely dominated by the salt ions K^+ and Cl^- since the NPs concentration is fM, which allows to simplify equation S1 to obtain equation S4.

$$t_{PS} = \frac{|z_{PS}| D_{PS} C_{PS}}{(D_{K^+} + D_{Cl^-}) C_{KCl}} \qquad \text{(S4)}$$

t_{ps} could also be obtained by the equation S5

$$t_{PS} = i_{PS} / i_{Total} \qquad \text{(S5)}$$

i_{Total} is the total current across the nanopipet (i.e., I).

The current of NPs can be converted to the flux of NPs by equation S6.

$$j_{mig} = i_{PS} / z_{PS} F \qquad \text{(S6)}$$

Considering the relationship of mobility and diffusion coefficient as shown in equation S7,

$$u_i = z_i D_i F / RT \qquad \text{(S7)}$$

One can estimate the flux of NPs migrating to the nanopipet orifice per second by equation S6.

$$j_{mig} = \frac{IC_{PS}}{FC_{KCl}} \frac{u_{PS}}{u_{K^+} + u_{Cl^-}} \qquad \text{(S6)}$$

The frequency of NPs is obtained simply by multiplying by Avogadro's number.

$$f_{mig} = N_A j_{mig} = \frac{IC_{PS}}{eC_{KCl}} \frac{u_{PS}}{u_{K^+} + u_{Cl^-}} \tag{S7}$$

The diffusion coefficient of PS particles is approximately calculated by equation S8.

$$D = \frac{\kappa T}{6\pi\eta r_{ps}} \tag{S8}$$

S7 *I-t* Trace at A Negative Potential

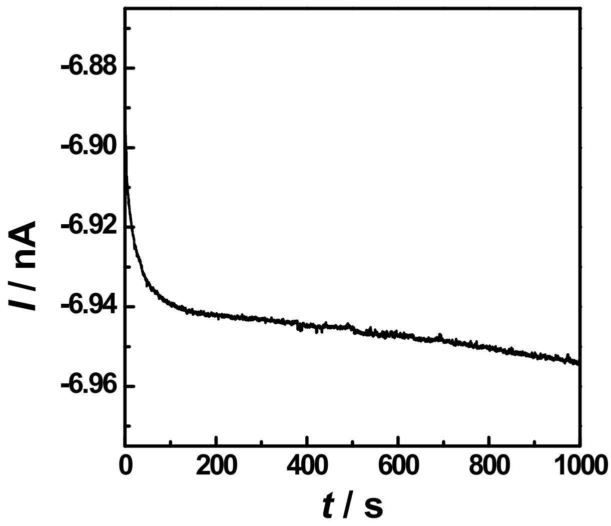

Fig. S8. *i-t* reponse for 16.9 fM 2.25-μm-radius PS particles suspended in 0.1 M KCl solution in the presence of 69-nm-radius nanopipet at -0.2 V. The data acquisition time was 10 ms.

S8 *I-t* Traces at Different Applied Potentials

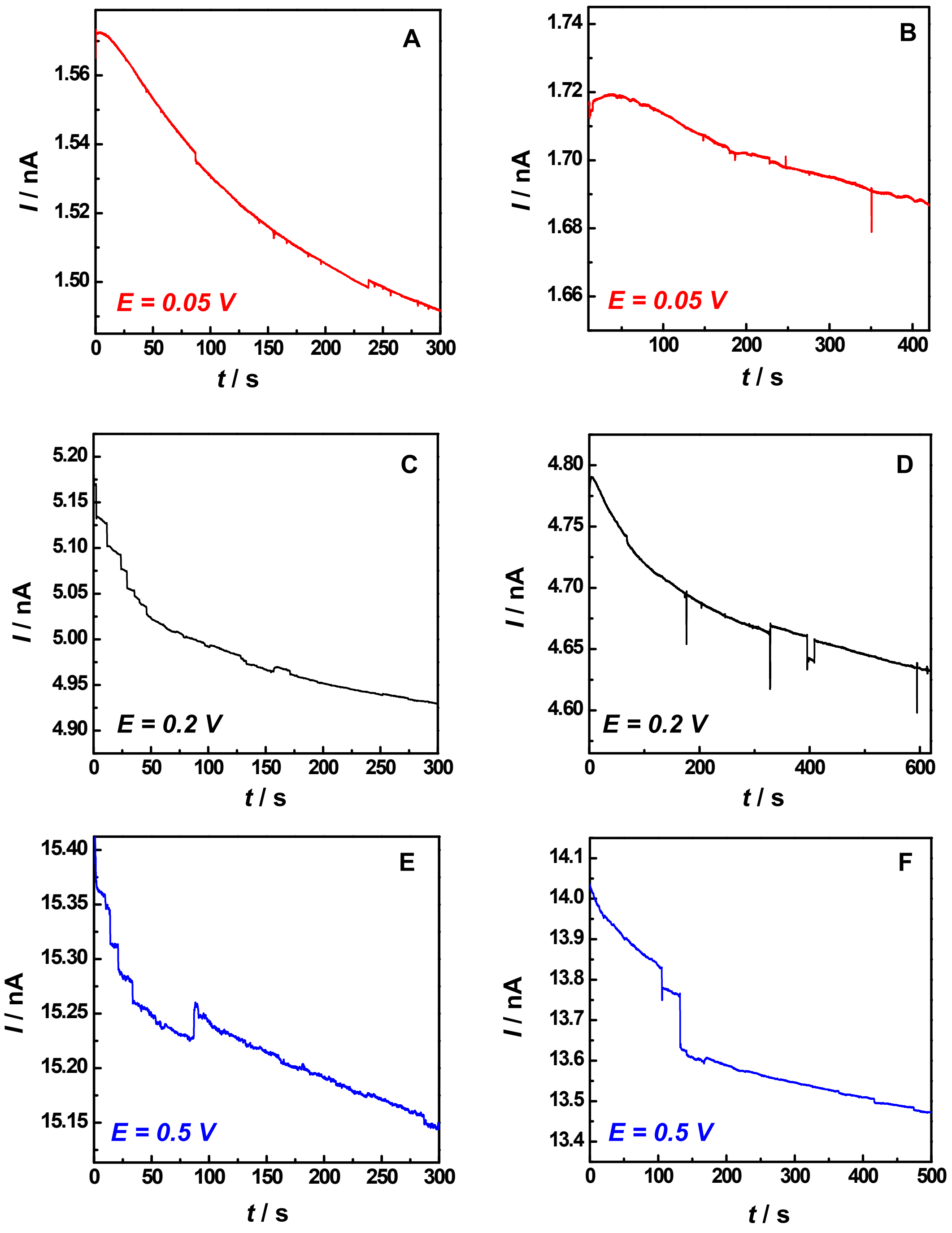

Fig. S9. *I-t* traces obtained from nanopipets with average radius as 69 nm in 0.1 M KCl solution containing 0.59 pM 375-nm-radius PS particles (A), (C), (D) and 16.9 fM 2.25-µm-radius PS particles (B), (D), (F) at different applied potentials, as indicated in the figures. The data acquisition time was 10 ms.

S9 Current Transients at Different Particle Concentrations

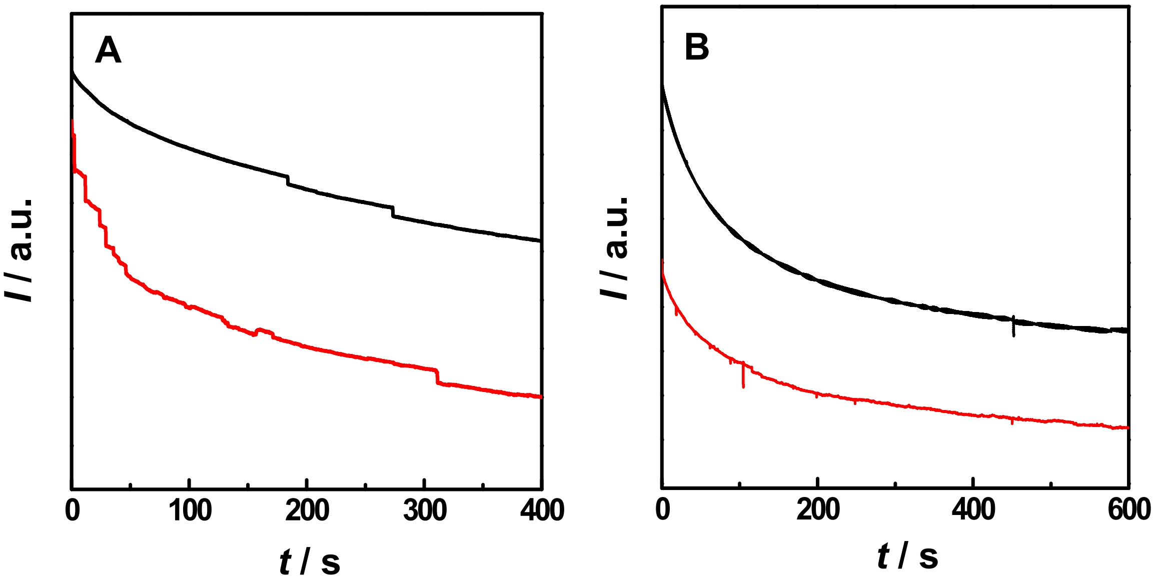

Fig. S10. *i-t* traces obtained from nanopipettes with average radius as 69 nm in 0.1 M KCl solution containing (A) 375-nm-radius PS particles with concentrations of 5.9 fM (black curve) and 590 fM (red curve) and (B) 2.25-µm-radius PS particles with concentrations of 3.38 fM (black curve) and 50.7 fM (red curve). The applied potential was 0.2 V. The data acquisition time was 10 ms.
